# Leveraging Open Electronic Health Record Data and Environmental Exposures Data to Derive Insights Into Rare Pulmonary Disease

**DOI:** 10.3389/frai.2022.918888

**Published:** 2022-06-28

**Authors:** Karamarie Fecho, Stanley C. Ahalt, Michael Knowles, Ashok Krishnamurthy, Margaret Leigh, Kenneth Morton, Emily Pfaff, Max Wang, Hong Yi

**Affiliations:** ^1^Renaissance Computing Institute, University of North Carolina at Chapel Hill, Chapel Hill, NC, United States; ^2^School of Medicine, University of North Carolina at Chapel Hill, Chapel Hill, NC, United States; ^3^CoVar Applied Technologies, Durham, NC, United States; ^4^North Carolina Clinical and Translational Sciences Institute, University of North Carolina at Chapel Hill, Chapel Hill, NC, United States

**Keywords:** open clinical data, rare disease, application programming interface, environmental exposures, environmental health, biomedical informatics

## Abstract

Research on rare diseases has received increasing attention, in part due to the realized profitability of orphan drugs. Biomedical informatics holds promise in accelerating translational research on rare disease, yet challenges remain, including the lack of diagnostic codes for rare diseases and privacy concerns that prevent research access to electronic health records when few patients exist. The Integrated Clinical and Environmental Exposures Service (ICEES) provides regulatory-compliant open access to electronic health record data that have been integrated with environmental exposures data, as well as analytic tools to explore the integrated data. We describe a proof-of-concept application of ICEES to examine demographics, clinical characteristics, environmental exposures, and health outcomes among a cohort of patients enriched for phenotypes associated with cystic fibrosis (CF), idiopathic bronchiectasis (IB), and primary ciliary dyskinesia (PCD). We then focus on a subset of patients with CF, leveraging the availability of a diagnostic code for CF and serving as a benchmark for our development work. We use ICEES to examine select demographics, co-diagnoses, and environmental exposures that may contribute to poor health outcomes among patients with CF, defined as emergency department or inpatient visits for respiratory issues. We replicate current understanding of the pathogenesis and clinical manifestations of CF by identifying co-diagnoses of asthma, chronic nasal congestion, cough, middle ear disease, and pneumonia as factors that differentiate patients with poor health outcomes from those with better health outcomes. We conclude by discussing our preliminary findings in relation to other published work, the strengths and limitations of our approach, and our future directions.

## Introduction

Rare diseases, while rare, collectively represent a large number of patients and place an enormous burden on healthcare systems, families, and caregivers. Drug discovery and drug repurposing for rare diseases have received increasing attention over the past decade, in part due to the realization that profits can be made from so-called orphan drugs, despite the relatively small market, and in part due to the advocacy of groups such as the International Rare Disease Consortium (Austin et al., 2018) and the National Organization for Rare Disorders (Dunkle, 2014). National Institutes of Health funding for rare disease and orphan drugs reflects this trend (National Institutes of Health., 2021). Advances in biomedical informatics promise to accelerate clinical and translational research, including research on rare diseases. Yet, many challenges remain, including regulatory and privacy concerns that hinder research access to the electronic health records (EHRs) of patients with rare disease when few patients exist within a healthcare system; the lack of definitive diagnostic codes for the majority of rare diseases; complexities related to data integration and semantic harmonization across disparate data sources; inconsistencies in the adoption of standardized ontologies, vocabularies, and terminologies; and domain-specific differences in terminologies and data representations (Wilkinson et al., 2016; Haixiang et al., 2017; Colbaugh et al., 2018; Shen et al., 2018; Cohen et al., 2020).

To begin to address these challenges, we have developed an open-source solution that can be used to explore EHR data on patients with rare diseases and identify factors that may contribute to health outcomes. Specifically, the Integrated Clinical and Environmental Exposures Service (ICEES) is an open service that exposes, in a regulatory-compliant manner, EHR data that have been integrated at the patient level with a variety of environmental exposures data (Fecho et al., 2019; Pfaff et al., 2019; Xu et al., 2020). ICEES also provisions analytic tools to explore the integrated data. As such, ICEES provides a powerful open-source, regulatory-compliant solution that allows users to readily explore real-world clinical observations, including observations related to rare disease, and conduct basic analyses designed to investigate environmental exposures and other factors that may influence health outcomes.

Herein, we describe a proof-of-concept application of ICEES to examine demographics, clinical characteristics, environmental exposures, and health outcomes among a cohort of patients enriched for phenotypes (i.e., diagnoses and procedures) associated with rare pulmonary diseases, namely, cystic fibrosis (CF), idiopathic bronchiectasis (IB), and primary ciliary dyskinesia (PCD). We focus on a subset of patients with CF, leveraging the availability of a diagnostic code for CF and serving as a benchmark for our development work. We use ICEES to examine select demographics, co-diagnoses, and environmental exposures that may contribute to poor health outcomes among patients with CF, defined as emergency department (ED) or inpatient visits for respiratory issues, with the goal of replicating prior work. We then discuss our findings in the context of published work, the strengths and limitations of our approach, and our future directions.

## Methods

All study procedures were approved by the Institutional Review Board at the University of North Carolina at Chapel Hill (protocol #21-0099).

### “Rare Pulmonary Disease” Cohort

Our overall goal was to define an ICEES “rare pulmonary disease” cohort to explore demographic factors, clinical characteristics, and environmental exposures that impact health outcomes. We focused on a 1-year study period (calendar year 2020) and the rare diseases CF, IB, and PCD (Orphanet rare disease resources, June 13, 2022). Given that a definitive diagnostic code was available only for CF (ICD-E84), we identified patients as having *possible* CF, IB, or PCD using broad, expert-defined inclusion criteria based on EHR data from UNC Health. Specifically, we included patients who met the following criteria: (1) hospital or clinic location: one or more visits to the adult pulmonary clinic, any pediatric clinic, the male infertility clinic, the general hospital inpatient service for adult patients, any hospital inpatient service for pediatric patients with CPT codes for respiratory therapy and/or physical therapy, or admission to the Neonatal Intensive Care Unit for term births (≥35 weeks gestation); (2) diagnosis or procedure: one or more diagnoses or procedures for congenital heart disease with situs inversus/laterality/dextrocardia/laterality defect, sinus surgery before age 6 years, tympanoplasty tubes before age 1 year, echocardiograms before age 6 months, Kartagener's syndrome (situs inversus), bronchiectasis, heterotaxy, semen analysis, or male infertility.

### ICEES

ICEES provides regulatory-compliant open access to binned, observational clinical data that have been integrated at the patient level with public environmental exposures data (Fecho et al., 2019). Key to the design of ICEES are “ICEES integrated feature tables.” These tables are created using a complex custom software pipeline within a secure environment and under a protocol approved by the Institutional Review Board at the University of North Carolina at Chapel Hill. In brief, a custom, open-source application, Clinical Asset Mapping Program for Health Level 7 Fast Healthcare Interoperability Resources (CAMP FHIR), is used to extract and convert patient EHR data from the PCORnet common data model to FHIR files (Pfaff et al., 2019). A second open-source application, FHIR Patient data Integration Tool (FHIR PIT), then ingests the FHIR files and integrates the patient data with public sources of environmental exposures data, using geocodes and dates (Xu et al., 2020). The final step in the FHIR PIT pipeline is the binning or recoding of each feature variable and the de-identification of the integrated data per the Safe Harbor method outlined in the Health Insurance Portability and Accountability Act (HIPAA). The final ICEES integrated feature tables are then deployed behind an open application programming interface (OpenAPI).

The public exposures data are derived from several sources: the United States (US) Environmental Protection Agency's Fused Air Quality Surface Using Downscaling repository on airborne pollutants; the US Department of Transportation's repository on roadway and highway exposures (a proxy for airborne pollutant exposures); the US Census Bureau's American Community Survey data on socio-economic exposures; and the North Carolina Department of Environmental Quality's repository on landfills and concentrated animal feeding operations (CAFOs). The availability of these public sources of exposures data depends on the year of interest. In this study, we focused on the year 2020 as the study period of interest and exposure estimates for residential density and distance between primary residence and nearest major roadway or highway, landfill, or CAFO. Distance estimates were based on patient primary residential address, as listed in the EHR. (Additional information on the sources of environmental exposures data can be found in Fecho et al., 2019; Valencia et al., 2020).

The binning or recoding step in the ICEES pipeline is necessary per institutional mandate to abstract the data and thereby create an added layer of privacy beyond HIPAA Safe Harbor deidentification. This mandate largely reflects our institution's position that any data derived using protected health information (PHI) such as exposure estimates are treated as “secondary PHI” and therefore subject to privacy regulations. We based our binning or recoding approach on a combination of published models, subject matter expert recommendation, and mathematical algorithms (Fecho et al., 2019). In brief, ages were calculated from day one of the 1-year study period and binned as <5, 5–17, 18–44, 45–64, 65–89 years, per our prior work (Fecho et al., 2008a,b, 2019), with 89 years treated as the ceiling per HIPAA. Sex was categorized as biological male or female, as listed in the EHR. Race was categorized as Caucasian, African American, Asian, and Other. Ethnicity was categorized as Hispanic or Latino vs. not Hispanic or Latino. Diagnoses were based on diagnostic codes and treated as binary (0, no; 1, yes), calculated as: 0, 1, >1 diagnoses over the 1-year study period. Residential density was based on the US Census Bureau's classification: rural area (<2,500 persons per Census block group); urban cluster (2,500 up to 50,000 persons per Census block group); and urbanized area (>50,000 persons per Census block group). Proximity from primary residence to a major roadway or highway was binned based on published work (Schurman et al., 2018): 0–49, 50–99, 100–149, 150–199, 200–249, ≥250 meters). Distance between primary residence and nearest landfill or CAFO was modeled using a combination of subject matter expert recommendation and published modeling approaches (Radon et al., 2007; Rasmussen et al., 2017; Njoku et al., 2019; Tomita et al., 2020): <500, 500–1,000, 1,000–2,000, 2,000–4,000, >4,000 meters.

### Analytic Approach

In this study, we first queried ICEES to examine demographics, diagnoses, environmental exposures, and health outcomes among patients within the rare pulmonary disease cohort with possible CF, IB, or PCD. As noted above, ages were based on day one of the 1-year study period (calendar year 2020); diagnoses were based on one or more diagnostic codes for a condition over the 1-year study period; and environmental exposures were based on estimated residential density and nearest distance from primary residence to major roadway or highway, landfill, or CAFO.

We then used ICEES to create a subcohort of patients with one or more diagnostic codes for CF (ICD-E84) and an active EHR over the 1-year study period, meaning that the patient was seen by a UNC Health provider at least one time during the study period. Our primary health outcome was ED or inpatient visits for any respiratory issue, as defined in Fecho et al. (2019). We applied the ICEES multiple comparison functionality to identify demographic factors, co-diagnoses, and environmental exposures that differ between patients with CF and poor health outcomes, defined as one or more ED or inpatient visits for respiratory issues, and patients with CF and better health outcomes, defined as zero ED or inpatient visits for respiratory issues. We applied a Chi Square test with multiple comparisons and a Bonferroni-corrected α = 0.05.

## Results

### Characterization of Rare Pulmonary Disease Cohort

We first queried ICEES to examine the characteristics of the broadly defined rare pulmonary disease cohort (*N* = 4,840) (Table 1).

**Table 1:** Demographics, co-diagnoses, and environmental exposures among patients within the ICEES rare pulmonary disease cohort (*N* = 4,840).

**Characteristic**	***n* (%)**
**Demographics**, ***N*** **=** **4,840**	
Age^a^	
<5	270 (5.58)
5–17	396 (8.18)
18–44	702 (14.50)
45–64	1,106 (22.85)
65–89	2,366 (48.88)
Sex	
Male	1,931 (39.90)
Female	2,909 (60.01)
Race and ethnicity^b^	
Caucasian	
Hispanic or Latino	47 (0.97)
Not Hispanic or Latino	3,361 (69.44)
Missing	21 (0.43)
African American	
Hispanic or Latino	<10 (ND)
Not Hispanic or Latino	921 (19.03)
Missing	<10 (ND)
Asian	
Hispanic or Latino	<10 (ND)
Not Hispanic or Latino	77 (1.59)
Missing	<10 (ND)
**Diagnoses^c^**, ***N*** **=** **4,840**	
Anxiety	
0	4,133 (85.39)
1	89 (1.84)
>1	618 (12.77)
Asthma	
0	4,188 (85.08)
1	110 (2.27)
>1	612 (12.64)
Bronchiectasis	
0	3,832 (79.17)
1	150 (3.10)
>1	858 (17.73)
Chronic nasal congestion	
0	4,737 (97.87)
1	53 (1.10)
>1	50 (1.03)
Chronic obstructive pulmonary disease	
0	4,048 (83.64)
1	124 (2.56)
>1	668 (13.80)
Congenital malformation of respiratory system	4,840 (100.00)
0	0 (0)
1	0 (0)
>1	
Cough	
0	4,016 (82.98)
1	258 (5.33)
>1	566 (11.69)
Croup	
0	4,836 (99.92)
1	3 (0.06)
>1	1 (0.02)
Cystic fibrosis	
0	4,677 (96.63)
1	8 (0.17)
>1	155 (3.20)
Depression	
0	4,201 (86.80)
1	73 (1.51)
>1	566 (11.69)
Diabetes	
0	4,109 (84.9)
1	58 (1.20)
>1	673 (13.90)
Middle ear disease	
0	4,687 (96.84)
1	60 (1.24)
>1	93 (1.92)
Neonatal respiratory distress	
0	4,839 (99.98)
1	1 (0.02)
>1	0 (0)
Obesity	
0	4,362 (90.12)
1	65 (1.34)
>1	413 (8.53)
Pneumonia	
0	4,438 (91.69)
1	69 (1.43)
>1	333 (6.88)
Reactive airway disease	
0	4,048 (83.64)
1	124 (2.56)
>1	668 (13.80)
Situs inversus or heterotaxy	
0	4,736 (97.85)
1	39 (0.81)
>1	65 (1.34)
**Environmental Exposures^d^**, ***N*** **=** **1,327**	
CAFO (distance from primary residence, meters)	
<500	7 (0.53)
500–1,000	16 (1.21)
1,000–2,000	69 (5.20)
2,000–4,000	172 (12.96)
>4,000	1,063 (80.11)
Landfill (distance from primary residence, meters)	
<500	0 (0.00)
500–1,000	6 (0.45)
1,000–2,000	22 (1.66)
2,000–4,000	75 (5.65)
>4,000	1,224 (92.24)
Major roadway or highway (distance from primary residence, meters)	
0–49	228 (17.18)
50–99	72 (5.43)
100–149	92 (6.93)
150–199	95 (7.16)
200–249	86 (6.48)
≥250 meters	754 (56.82)
Residential density (persons per US Census block group)	
Rural <2500	894 (67.37)
Urban cluster ≥2,500–50,000	419 (31.57)
Urbanized area ≥50,000	0 (0.00)
Missing	14 (1.06)

*EHR, electronic health record; HIPAA, health insurance portability and accountability act; ICD, International Classification of Diseases; ND, not determined per HIPAA*.

a *Ages were calculated based on day one of the one-year study period (calendar year 2020)*.

b *Race and ethnicity were abstracted directly from patient HER*.

c *Diagnoses were based on ICD codes in the EHR and treated as 0, 1, or >1 diagnoses over the 1-year study period*.

d *Environmental exposures were based on primary residence, as documented in the HER*.

The demographic profile indicated that most patients were middle age or older [5.58% (270/4,840) <5 years, 8.18% (396/4,840) 5–17 years, 14.50% (702/4,840) 18–44 years, 22.85% (1,106/4,840) 45–64 years, 48.88% (2,366/4,840) 65–89 years)], female [60.01% (2,909/4,840)], and non-Hispanic/Latino Caucasian [69.44% (3,361/4,840)] or African American [19.03% (921/4,840)].

We also examined select diagnoses based on their relevance to CF, IB, and PCD. Over the 1-year study period, ten percent or more of patients had one or more diagnoses for anxiety [14.61% (707/4,840)], asthma [14.92% (722/4,840)], bronchiectasis [20.83% (1,008/4,840)], chronic obstructive pulmonary disease [16.36% (792/4,840)], cough [17.02% (824/4,840)], depression [13.20% (639/4,840)], diabetes [15.10% (731/4,840)], or reactive airway disease [16.36% (792/4,840)].

Environmental exposure estimates were calculated for those patients with valid geocodes and time stamps [27.42% (1,327/4,840)]. The majority of patients had a primary residence that was >4,000 meters from a CAFO [1,063 (80.11%)] or a landfill [92.24% (1,224/1,327)], with few patients residing <500 meters from a CAFO [0.53% (7/1,327)] or a landfill [0.00% (0/1,327)]. In addition, patients tended to reside either <50 meters or ≥250 meters from a major roadway or highway [17.18% (228/1,327) 0–49 meters, 5.43% (72/1,327) 50–99 meters, 6.93% (92/1,327) 100–149 meters, 7.16% (95/1,327) 150–199 meters, 6.48% (86/1,347) 200–249 meters, 56.82% (754/1,327) ≥250 meters] and within a region classified by the US Census Bureau as rural [67.37% (894/1,327)] or urban cluster [31.57% (419/1,327)]. No patients (0.00%) resided in a region classified as urbanized.

### Health Outcomes Among Patients Within the Rare Pulmonary Disease Cohort

**Figure 1:**
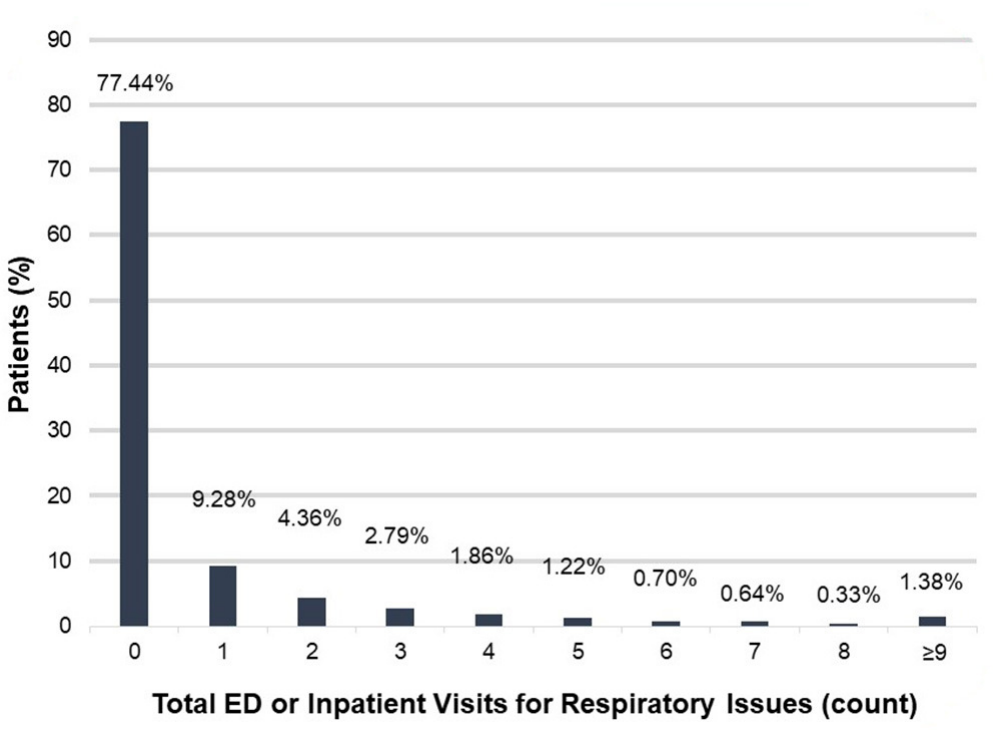
Total ED or inpatient visits for respiratory issues among patients within the rare pulmonary disease cohort over the 1-year study period. ED, emergency department.

We then used ICEES to examine health outcomes for patients within the rare pulmonary disease cohort. We focused on ED or inpatient visits for respiratory issues over the 1-year study period (Figure 1). The majority of patients [77.44% (3,748/4,840)] did not have any ED or inpatient visits for respiratory issues. Of those who had at least one ED or inpatient visit for respiratory issues, 9.28% (449/4,840) had one visit, 4.36% (211/4,840) had two visits, 2.79% (135/4,840) had three visits, 1.86% (90/4,840) had four visits, 1.22% (59/4,840) had five visits, 0.70% (34/4,840) had six visits, 0.64% (31/4,840) had seven visits, 0.33% (16/4,840) had eight visits, and 1.38% (67/4,840) had nine or more visits. The maximum number of ED or inpatient visits for respiratory issues over the 1-year study period was 35.

### Health Outcomes Among Patients With CF

We next used ICEES to examine health outcomes among the 163 patients (3.37% (163/4,840)) with a diagnosis of CF. Thirty-seven patients with CF (22.70%) had one or more visits to the ED or an inpatient clinic for respiratory issues over the 1-year study period (maximum = 8) (Table 2).

**Table 2:** Factors influencing health outcomes among patients with CF^a^ (*N* = 163).

**Characteristic**	**Total ED/inpatient visits = 0, *N* = 126, *n* (row %)**	**Total ED/inpatient visits ≥1, *N* = 37, *n* (row %)**	**χ^2^, *P-*value^b^**
**Demographics**, ***N*** **=** **163**
Age (years)			
<5	7 (100.00)	0 (0.00)	χ^2^ = 5.3851, *P* = 0.2500
5–17	40 (70.18)	17 (29.82)	
18–44	63 (80.77)	15 (19.23)	
45–64	13 (72.22)	5 (27.78)	
65–89	3 (100.0)	0 (0.00)	
Sex			
Male	66 (80.49)	16 (19.51)	χ^2^ = 0.9553, *P* = 0.3284
Female	60 (74.07)	21 (25.93)	
Race/ethnicity^c^	ND	ND	ND
**Co-diagnoses**, ***N*** **=** **163**
Anxiety			
0	88 (78.57)	24 (21.43)	χ^2^ = 0.4247, *P* = 0.8087
1	4 (80.00)	1 (20.00)	
>1	34 (73.91)	12 (26.09)	
Asthma			
0	108 (94.74)	6 (5.26)	χ^2^ = 66.5946, ***P*** **<** **0.0001**
1	1 (20.00)	4 (80.00)	
>1	17 (38.64)	27 (63.36)	
Bronchiectasis			
0	98 (79.67)	25 (20.33)	χ^2^ = 2.8042, *P* = 0.2461
1	6 (85.71)	1 (14.29)	
>1	22 (66.67)	11 (33.33%)	
Chronic nasal congestion			
0	119 (78.81)	32 (21.19)	χ^2^ = 6.6135, ***P*** **=** **0.03663**
1	3 (100.00)	0 (0.0)	
>1	4 (44.44)	5 (55.56)	
Chronic Obstructive Pulmonary Disease			
0	124 (77.50)	36 (22.50)	χ^2^=1.1468, *P*=0.5636
1	1 (50.00)	1 (50.0)	
>1	1 (100.0)	0 (0.00)	
Congenital malformation of respiratory system			
0	126 (77.30)	37 (22.70)	χ^2^ = 3.7737e−16, *P* = 1.00
1	0 (0.00)	0 (0.00)	
>1	0 (0.00)	0 (0.00)	
Cough			
0	108 (85.04)	19 (14.96)	χ^2^ = 21.0553, ***P*** **<** **0.0001**
1	10 (58.82)	7 (41.18)	
>1	8 (42.11)	11 (57.89)	
Croup			
0	126 (77.30)	37 (22.70)	χ^2^ = 3.7727e−16, *P* = 1.00
1	0 (0.00)	0 (0.00)	
>1	0 (0.00)	0 (0.00)	
Depression			
0	93 (76.86)	28 (23.14)	χ^2^ = 0.3201, *P* = 0.8521
1	1 (100.0)	0 (0.00)	
>1	32 (78.05%)	9 (21.95)	
Diabetes			
0	101 (80.16)	25 (19.84)	χ^2^ = 2.9560, *P* = 0.2281
1	1 (50.00)	1 (50.00)	
>1	24 (68.57)	11 (31.43)	
Male infertility			
0	126 (77.30)	37 (22.70)	χ^2^ = 3.7727e−16, *P* = 1.00
1	0 (0.00)	0 (0.00)	
>1	0 (0.00)	0 (0.00)	
Middle ear disease			
0	125 (79.11)	33 (20.89)	χ^2^ = 9.9371, ***P*** **=** **0.00169**
1	0 (0.00)	1 (100.00)	
>1	1 (25.00)	3 (75.00)	
Neonatal respiratory distress			
0	126 (77.78)	36 (22.22)	χ^2^=3.4264, *P*=0.1803
1	0 (0.00)	1 (100.0)	
>1	0 (0.00)	0 (0.00)	
Obesity			
0	124 (77.99)	35 (22.01)	χ^2^ = 1.7418, *P* = 0.4186
1	0 (0.00)	0 (0.00)	
>1	2 (50.00)	2 (50.00)	
Pneumonia			
0	115 (87.79)	16 (12.21)	χ^2^ = 41.8167, ***P** **<** **0.001***
1	2 (33.33)	4 (66.67)	
>1	9 (34.62)	17 (65.38)	
Reactive airway disease			
0	124 (77.50)	36 (22.50)	χ^2^ =1.1468, *P* = 0.5636
1	1 (50.00)	1 (50.00)	
>1	1 (50.00)	0 (0.00)	
Situs inversus or heterotaxy			
0	126 (77.30)	37 (22.70)	χ^2^ = 3.7737e−16, *P* = 1.00
1	0 (0.00)	0 (0.00)	
>1	0 (0.00)	0 (0.00)	
**Environmental exposures**, ***N*** **=** **42**
CAFO (distance from primary residence, meters)			
<500	1 (100.0)	0 (0.00)	χ^2^ = 2.0952, *P* = 0.8358
500–1,000	0 (0.00)	0 (0.00)	
1,000–2,000	1 (100.0)	0 (0.00)	
2,000–4,000	2 (40.0)	3 (60.00)	
>4,000	19 (54.29)	16 (45.71)	
Landfill (distance from primary residence, meters)			
<500	0 (0.00)	0 (0.00)	χ^2^ = 3.6522, *P* = 0.6005
500–1,000	0 (0.00)	0 (0.00)	
1,000–2,000	0 (0.00)	0 (0.00)	
2,000–4,000	4 (100.00)	0 (0.00)	
>4,000	19 (50.00)	19 (50.00)	
Major roadway or highway (distance from primary residence, meters)			
0–49	4 (57.14)	3 (42.86)	χ^2^ = 6.1606, *P* = 0.2909
50–99	1 (50.00)	1 (50.00)	
100–149	4 (57.14)	3 (42.86)	
150–199	3 (100.00)	0 (0.00)	
200–249	0 (0.00)	3 (100.00)	
≥250 meters	11 (55.00)	9 (45.00)	
Residential density (persons per US Census block group)			
Rural <2,500	15 (46.88)	17 (53.12)	χ^2^ = 3.3747, *P* = 0.1850
Urban cluster ≥2,500–50,000	8 (80.00)	2 (20.00)	
Urbanized area ≥50,000	0 (0.00)	0 (0.00)	

*ED, emergency department; EHR, electronic health record; ICD, International Classification of Diseases*.

a *Based on ICD code (ICD-Q84) in patient EHR, calendar year 2020*.

b *P values were calculated using Chi Square tests with Bonferroni-corrected α = 0.05. Significant values are indicated with bold font*.

c *Race and ethnicity were not included in the analysis*.

The demographic composition of patients with CF who had poor health outcomes was similar to that among patients with CF and better health outcomes. Environmental exposures were likewise similar among patients with CF, regardless of health outcome. However, several co-diagnoses differentiated patients with CF and poor health outcomes from those with better health outcomes.

**Figure 2:**
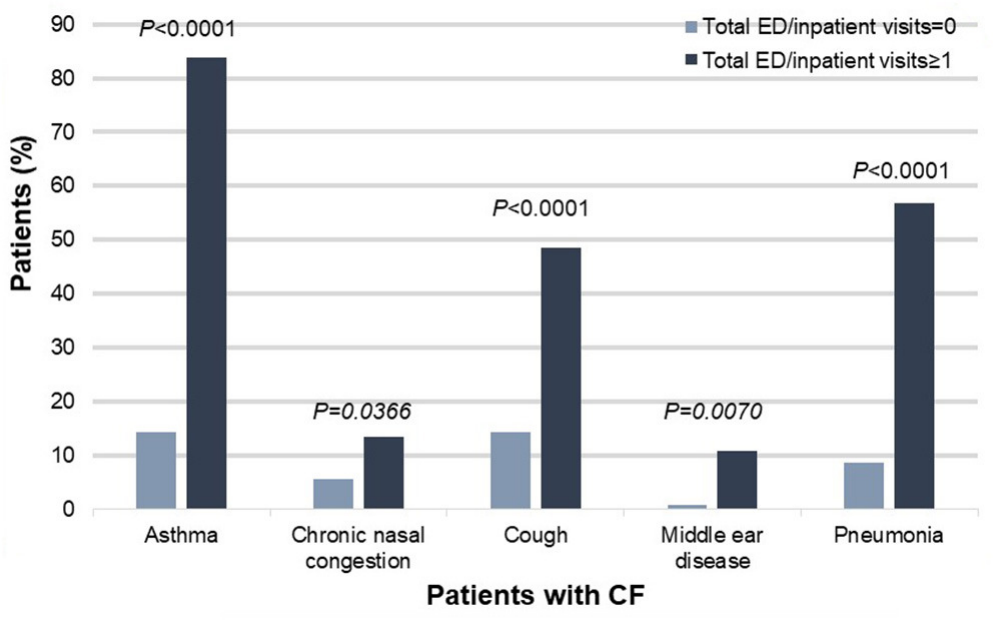
Co-diagnoses that significantly differed between patients with CF and poor health outcomes (defined as one or more ED or inpatient visits for respiratory issues) vs. patients with CF and better health outcomes (defined as zero ED or inpatient visits for respiratory issues), *N* = 163, *P* < 0.05. CF, cystic fibrosis; ED, emergency department.

Specifically, patients with CF and poor health outcomes were more likely than those with better outcomes to have co-diagnoses of asthma [83.78% (31/37) vs. 14.29% (18/126), χ^2^ = 66.5946, *P* < 0.0001], chronic nasal congestion [13.51% (5/37) vs. 5.56% (7/126), χ^2^= 6.61354, *P* = 0.0366], cough [48.65% (18/37) vs. 14.29% (18/126), χ^2^ = 21.0553, *P* < 0.0001], middle ear disease [10.81% (4/37) vs. 0.79% (1/126), χ^2^ = 9.9371, *P* = 0.00169], and pneumonia [56.76% (21/37) vs. 8.73% (11/126), χ^2^ = 41.8167, *P* < 0.001] (Figure 2).

## Discussion

### Summary of Findings and Relationship to Other Published Work

In this study, we defined a cohort of patients enriched for phenotypes (i.e., diagnoses and procedures) associated with rare pulmonary diseases, specifically, CF, IB, and PCD. We deployed the dataset behind an ICEES OpenAPI and used ICEES to examine demographics, clinical characteristics, environmental exposures, and health outcomes among patients within the cohort. We then used ICEES to focus on a subset of patients with a diagnostic code for CF and applied the ICEES multiple comparisons functionality to determine factors that significantly differed among patients with CF and poor health outcomes vs. those with better outcomes. We were able to replicate current understanding of the pathogenesis and clinical manifestations of CF (e.g., Turcios, 2020) by identifying several co-diagnoses that differentiated patients with CF and poor health outcomes from those with better outcomes: asthma; chronic nasal congestion; cough; middle ear disease; and pneumonia. Demographic factors and environmental exposures were not associated with health outcomes among patients with CF.

Several findings and other points are worth discussing in relation to their interpretation and previously published work. For instance, the inclusion criteria that we used to define the ICEES rare pulmonary disease cohort were intentionally broad, the goal being to create a dataset enriched in phenotypes associated with *possible* CF, IB, and PCD rather than definitive diagnostic codes, which do not exist for IB and PCD. The intent behind our broad inclusion criteria was to create a rare pulmonary disease dataset with multiple applications, including the one reported here and others such as enabling expert chart review to determine *definitive* diagnoses of CF, IB, and PCD and providing a training dataset to support supervised machine learning estimates of *predicted* diagnoses of CF, IB, and PCD. We have moved forward with both additional applications and plan to incorporate the definitive diagnoses and predictions into the next deployment of the ICEES rare pulmonary disease OpenAPI.

While EHR data are intended to support healthcare administration and billing, not research, such records provide a valuable source of research data that, when used appropriately, can accelerate clinical and translational research, including research on rare diseases. For instance, classification and machine learning models have been developed and successfully applied by our group (Pfaff et al., 2020) and others (Colbaugh et al., 2018; Shen et al., 2018; Cohen et al., 2020) to leverage available EHR data in a subject matter expert–informed manner and use that collective information to predict patients who have specific rare diseases. Moreover, to support the application of EHR data for research, numerous statistical techniques and software packages have been developed to account for the missing data and imbalances that are inherent in EHR data (e.g., Chawla et al., 2002; Wells et al., 2013; Haixiang et al., 2017). ICEES extends EHR data to include environmental exposures data and exposes the integrated data for open exploratory analysis. While the present proof-of-concept study did not identify significant environmental impacts on health outcomes among patients with CF, our prior work has (e.g., Fecho et al., 2019, 2022), and we expect the production version of the ICEES rare pulmonary disease instance to likewise reveal clinical and environmental influences on health outcomes.

Another consideration is that the co-diagnoses that we identified as related to poor health outcomes among patients with CF are all pulmonary and perhaps not unexpected. This reflects several factors. First, we considered the initial ICEES rare pulmonary disease instance as a proof-of-concept application of ICEES to the study of rare disease, and so, we focused on a relatively small subset of EHR data. In addition, we did not capture medications, laboratory measures, or procedures, which we have done for other ICEES cohorts. The CAMP FHIR/FHIR PIT data conversion and integration software pipeline relies on an enumeration file that is manually generated. The enumeration file that supports the ICEES PCD instance does not currently support medications, laboratory measures, or procedures. Having demonstrated proof of concept, we are now updating the file and correcting technical bugs in our software pipeline that were detected as part of the work described herein. We expect to continuously improve and extend the ICEES rare pulmonary disease instance, thus providing a unique open-source resource for exploratory analysis of clinical and environmental determinants of health.

A related point to consider is that the co-diagnoses we identified as affecting health outcomes among patients with CF may or may not represent comorbidities. The study design and available data did not allow us to differentiate between patients who simply became ill over the study period vs. those with chronic comorbidities.

Our findings on environmental exposures that differentiate patients with CF and poor health outcomes from those with better outcomes are also worth discussion. Specifically, we did not identify a relationship between proximity to a landfill or CAFO and poor health outcomes among patients with CF. In fact, most patients with CF and poor health outcomes resided >4,000 meters from a landfill or CAFO. These findings contradict published findings on the pulmonary effects of landfill and CAFO exposures (e.g., Radon et al., 2007; Rasmussen et al., 2017; Njoku et al., 2019; Tomita et al., 2020). Likewise, we did not find a relationship between proximity to a major roadway or highway and health outcomes among patients with CF, which contradicts prior findings on asthma exacerbations from our group (Fecho et al., 2022) and other groups (Perez et al., 2012; Schurman et al., 2018; Hauptman et al., 2020). Finally, we did not identify a relationship between rural residence and poor health outcomes among patients with CF, which contradicts our prior findings on asthma exacerbations (Fecho et al., 2022) and the well-established rural health disparities in North Carolina and elsewhere (North Carolina Institute of Medicine, 2014).

We believe that there are several explanations for these apparent discrepancies. First, our patient catchment area is largely rural, as we have reported previously (e.g., Fecho et al., 2022). As such, the current findings may simply reflect an inherent bias in our data that may have obscured rural health disparities in health outcomes in the current study. Second, our geocoding was sparse. In fact, only 27.42% of patients were successfully geocoded, unlike our prior work, in which few patients lacked valid geocodes (e.g., Fecho et al., 2019; Xu et al., 2020). The sparse geocoding in the current effort reflects a change in our hospital's geocoding practices that introduced errors, since resolved. Third, our models for estimating landfill and CAFO exposures may need refinement. This is the first study in which we applied landfill and CAFO exposures to patient data as part of ICEES. We are considering several more sophisticated models for landfill and CAFO exposures (Bunton et al., 2007; Son et al., 2021). We are also working with groups such as the Environmental Health Language Collaborative (National Institute of Environmental Health Sciences., 2021) to standardize and harmonize environmental health languages, ontologies, and exposure models. Finally, it may be that the environmental exposures that influence health outcomes among patients with CF and rare pulmonary diseases, or the models that we applied to estimate those exposures, differ from those that influence health outcomes among patients with asthma and common pulmonary diseases. We plan to expand the current work to include data on machine learning predictions and expert-confirmed diagnoses of CF, IB, and PCD, which will allow us to refine our exposure models and estimates and further explore the impact of environmental exposures on rare pulmonary diseases.

### Limitations

This study has several limitations, in addition to those discussed above, that should be considered when interpreting the results. First, our study focused on the year 2020, which limited the sources of environmental exposures data that we had access to. For example, public data on airborne pollutant exposures such as particulate matter were not available for year 2020. This is an ongoing challenge with ICEES, one that is largely out of our control. However, we continue to monitor public sources of environmental exposures data for new releases. Second, we did not capture certain clinical phenotypes of interest to our subject matter experts, including male infertility due to sperm tail dysfunction, low nasal nitric oxide levels, and defective ciliary ultrastructure. ICEES currently is limited to demographic data, diagnoses, medications (prescriptions or administrations), select laboratory measures and procedures, and select environmental exposures. We are working to expand the laboratory measures and procedures that are captured by ICEES, which will support richer analyses. Third, ICEES currently supports open multivariate analytic approaches such as logistic regression (Fecho et al., 2021). However, the approach introduces a certain amount of data loss and thus influences multivariate model robustness due to regulatory constraints surrounding small sample sizes. In the current study, the sample size for patients with CF was simply too small to invoke the ICEES multivariate approach, even without the data loss that the open multivariate approach introduces (Bujang et al., 2018). Small sample sizes are a challenge for research on rare disease. However, as demonstrated with this study, ICEES can be applied to gain insights into clinical and environmental determinants of health even with small sample sizes. Ours proof-of-concept demonstration study revealed the need for additional analytic features, including Fisher's Exact Test, to adjust for small cell sizes, as well as more sophisticated analytics to support clustering, enrichment analysis, and machine learning algorithms. We are developing approaches to implement new analytic features and expose them to users in an open regulatory-compliant manner. A final consideration, but not necessarily a limitation, is that ICEES is under continual development. As such, users should be aware that the service continues to evolve, with new feature variables and functionalities introduced over time. Users can access ICEES through the ICEES OpenAPI and associated Swagger user interface and are encouraged to post any issues that are identified in the ICEES GitHub repository (see References list for URLs).

## Conclusions

Here, we demonstrate the application of an open clinical service, ICEES, to explore clinical and environmental determinants of rare pulmonary disease. We focus on a subset of patients with a diagnosis of CF, and we replicate current understanding of CF by identifying co-diagnoses that differentiate patients with poor health outcomes from those with better health outcomes. ICEES was able to replicate prior findings without the need for regulatory approvals, patient recruitment, or complex epidemiological study design. As an open, regulatory-compliant, disease-agnostic service, ICEES has applications in the study of rare disease and can be used to overcome regulatory challenges and accelerate research. Moreover, ICEES has broader applications as a tool to inform public health research and delivery of health care, for example, by allowing researchers and healthcare providers to quickly explore the impact of clinical factors and environmental exposures on health outcomes, including patients with suspected rare disease, as was demonstrated in the work described herein.

## Data Availability Statement

The raw datasets presented in this article are not readily available because the data include sensitive patient data; rather, the data are available in a form consumable by the public *via* the ICEES rare pulmonary disease OpenAPI (see References list for URL).

## Ethics Statement

All study procedures were reviewed and approved by Institutional Review Board at the University of North Carolina at Chapel Hill (protocol #21-0099). Written informed consent for participation was not required for this study in accordance with federal legislation and institutional requirements.

## Author Contributions

KF, SA, MK, AK, and ML conceived the study. KF contributed to the design of the ICEES rare pulmonary disease OpenAPI, tested all software implementations, conducted all analyses, and prepared the first manuscript draft. MK and ML provided clinical subject matter expertise. KM, MW, and HY served as technical leads, led software development, and deployment of the ICEES OpenAPI. MK, ML, and EP developed the inclusion criteria for selecting patients within the rare pulmonary disease cohort. EP co-led the design and implementation of the CAMP FHIR software application. All authors contributed to the study design, assisted with interpretation of the results, reviewed the first draft of the manuscript, and approved the final submission.

## Funding

This work was supported by funding from the National Center for Advancing Translational Sciences (award numbers OT2TR003430, UL1TR002489, UL1TR002489-03S4, and OT3TR002020).

## Conflict of Interest

KM and MW were employed by CoVar Applied Technologies. The remaining authors declare that the research was conducted in the absence of any commercial or financial relationships that could be construed as a potential conflict of interest.

## Publisher's Note

All claims expressed in this article are solely those of the authors and do not necessarily represent those of their affiliated organizations, or those of the publisher, the editors and the reviewers. Any product that may be evaluated in this article, or claim that may be made by its manufacturer, is not guaranteed or endorsed by the publisher.
